# Multiple cerebral infarctions due to calcified amorphous tumor growing rapidly from an antecedent infection and decreasing rapidly by detachment of fibrin and antithrombotic drugs: a case report

**DOI:** 10.1186/s12883-022-02918-5

**Published:** 2022-10-22

**Authors:** Motoya Kimura, Jun-Ichi Niwa, Hideaki Ito, Katsuhiko Matsuyama, Manabu Doyu

**Affiliations:** 1 Department of Neurology, Aichi Medical University, 1-1 Yazakokarimata, Nagakute-City, Aichi 480-1195 Japan; 2 Department of Pathology, Aichi Medical University, 1-1 Yazakokarimata, Nagakute-City, Aichi 480-1195 Japan; 3 Department of Cardiovascular Surgery, Aichi Medical University, 1-1 Yazakokarimata, Nagakute-City, Aichi 480-1195 Japan

**Keywords:** Calcified amorphous tumor, Multiple cerebral infarctions, Antecedent infection, Fibrin, Case report

## Abstract

**Background:**

Calcified amorphous tumor (CAT) of the heart is a rare non-neoplastic intracardiac mass, a calcium deposition surrounded by amorphous fibrous tissue, and possibly causes cerebral embolism. Even rarer is CAT associated with infection, and no CAT with antecedent infection has been reported to our knowledge. In addition, although some CAT in patients on hemodialysis has been reported to grow rapidly, no case has been reported on CAT that grew and diminished rapidly in a short period of time. Here, we report the case of an 82-year-old Japanese woman with normal renal function who developed multiple cerebral infarctions due to CAT that grew rapidly, associated with inflammation from an antecedent infection, and diminished rapidly by detachment of fibrin on the mass surface and antithrombotic drugs.

**Case presentation:**

The patient developed fever after dental treatment and found musical hallucination on the left ear worsened in degree and frequency. In a nearby clinic, she was treated with antibiotics, and her body temperature turned to normal in approximately 1 month. She presented to our hospital for workup on the worsened musical hallucination. Magnetic resonance imaging (MRI) showed multiple cerebral infarctions, and transthoracic echocardiography (TTE) revealed an immobile hyperechoic mass with an acoustic shadow arising from a posterior cusp of the mitral valve. CAT was suspected and treated with apixaban and aspirin. Follow-up MRI and TTE showed newly developed multiple cerebral infarctions and rapidly diminished CAT. Cardiac surgery was performed to resect the CAT. The pathological findings showed calcifications surrounded by amorphous fibrous tissue including fibrin, indicating CAT. The patient’s symptoms improved and no cerebral infarctions recurred in 4 months follow-up.

**Conclusion:**

Inflammation from an antecedent infection can cause CAT to grow rapidly. Fibrous tissue including fibrin may attach to the surface of CAT, resulting in multiple cerebral infarctions. Fibrous tissue may detach and disappear by antithrombotic drugs, leading to a rapid diminishment of CAT in size.

## Background

Calcified amorphous tumor (CAT) of the heart is a rare non-neoplastic cardiac tumor, a calcium deposition surrounded by amorphous fibrous tissue [1]. It is not life-threatening itself, yet it is found to cause embolization. A previous study has reported pulmonary or systemic embolization in 31% of their CAT cases [2], and another study has reported approximately 20 cases of cerebral infarctions from CAT [3].

Though it may result in serious consequences, much is still unknown on CAT. According to a systematic review of 42 published cases from 1997 to 2014, CAT is frequently associated with valvular disease (31%), end-stage renal disease (21%), mitral annular calcification (MAC) (14%), diabetes (14%), and coronary artery disease (12%) [2]. It may be associated with infection, but only 2 cases, to our knowledge, have been reported; and it remains uncertain whether infection was a cause or a complication of CAT [4, 5]. A case of CAT with an antecedent infection, which would give a clue to this question, has not been reported so far.

Another notable aspect of CAT is a rapid change in size. In several cases, CAT is reported to grow rapidly in a short period of time, presumably because of abnormal calcium metabolism due to renal dysfunction [6], inflammation associated with hemodialysis [7], and mobille CAT arising from MAC [8]. However, no case has been reported where CAT rapidly grew AND diminished in a short period of time. In addition, in most cases of rapidly growing CAT, the patients were on hemodialysis [6, 7, 9]; few, if any, patients with normal renal function have been reported.

Here, we report a first case of CAT associated with an antecedent infection, causing multiple cerebral infarctions in an elderly female patient with normal renal function. The case is also remarkable in the sense that CAT grew rapidly, supposedly due to the inflammation by an antecedent infection, and diminished rapidly, probably by detachment of fibrin and by antithrombotic drugs. We believe that this case may help elucidate the features of CAT for a better clinical management.

## Case presentation

**Fig. 1:**
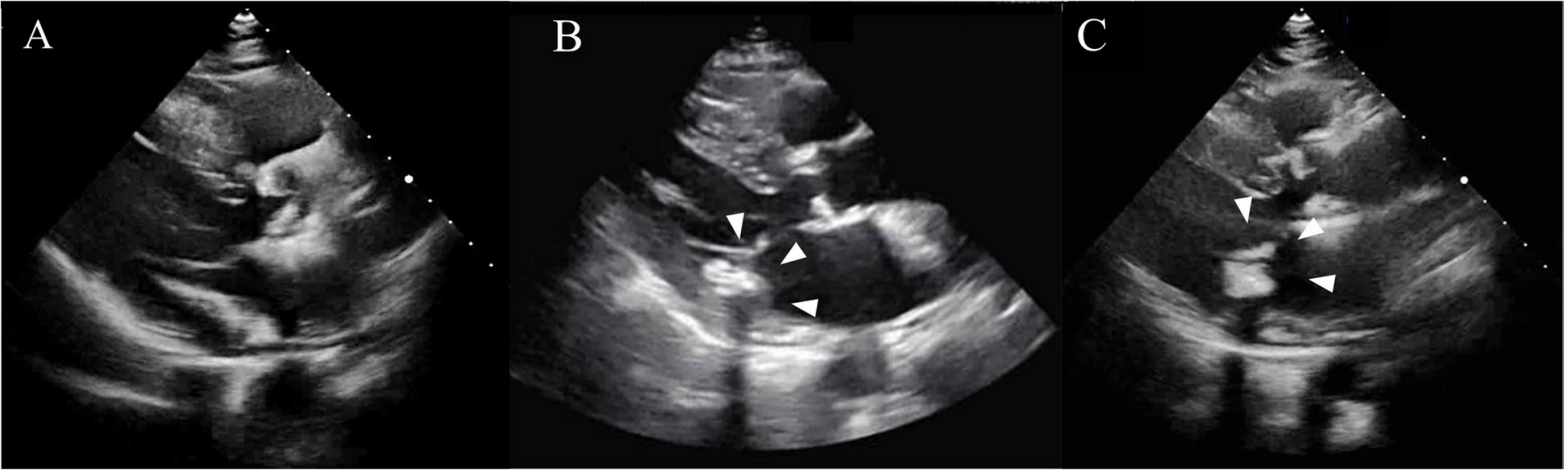
Serial transthoracic echocardiographs. A hyperechoic lesion or an acoustic shadow were not observed 7 months before the admission (**A**). An immobile hyperechoic 14.9 × 12.1 mm mass with an acoustic shadow arising from a posterior cusp of the mitral valve on admission (**B**, arrowheads). The mass was decreased to 13.8 × 9.7 mm in size on the 9 th day of admission (**C**, arrowheads)

An 82-year-old Japanese woman with bilateral hearing loss and musical hallucination was admitted to our hospital for transient ischemic attack (TIA) 3 times from 17 to 7 months before acute multiple cerebral infarctions occurred. Each time she underwent a workup including transthoracic echocardiography (TTE), which showed nothing appeared to be remarkable (Fig. 1A).

Six months later since the last admission, that is, 1 month before the infarctions, the patient saw a dentist for implant replacement. Starting the treatment, she developed fever over 38 °C. In a few days, she also noticed the degree and frequency of musical hallucination worsened in the left ear. She visited a nearby clinic, complaining of continuous fever, chill, and loss of appetite. Laboratory findings there showed elevated levels of 12,600 /μL in white blood cell count and 1.37 mg/dL in C-reactive protein. A whole body computed tomography scan, urinalysis, and chest X-ray were unremarkable. 2 separate sets of blood cultures were negative. Ceftriaxone and levofloxacin were administered, and the body temperature turned to normal in 1 month, whereas the worsened musical hallucination persisted.

**Fig. 2:**
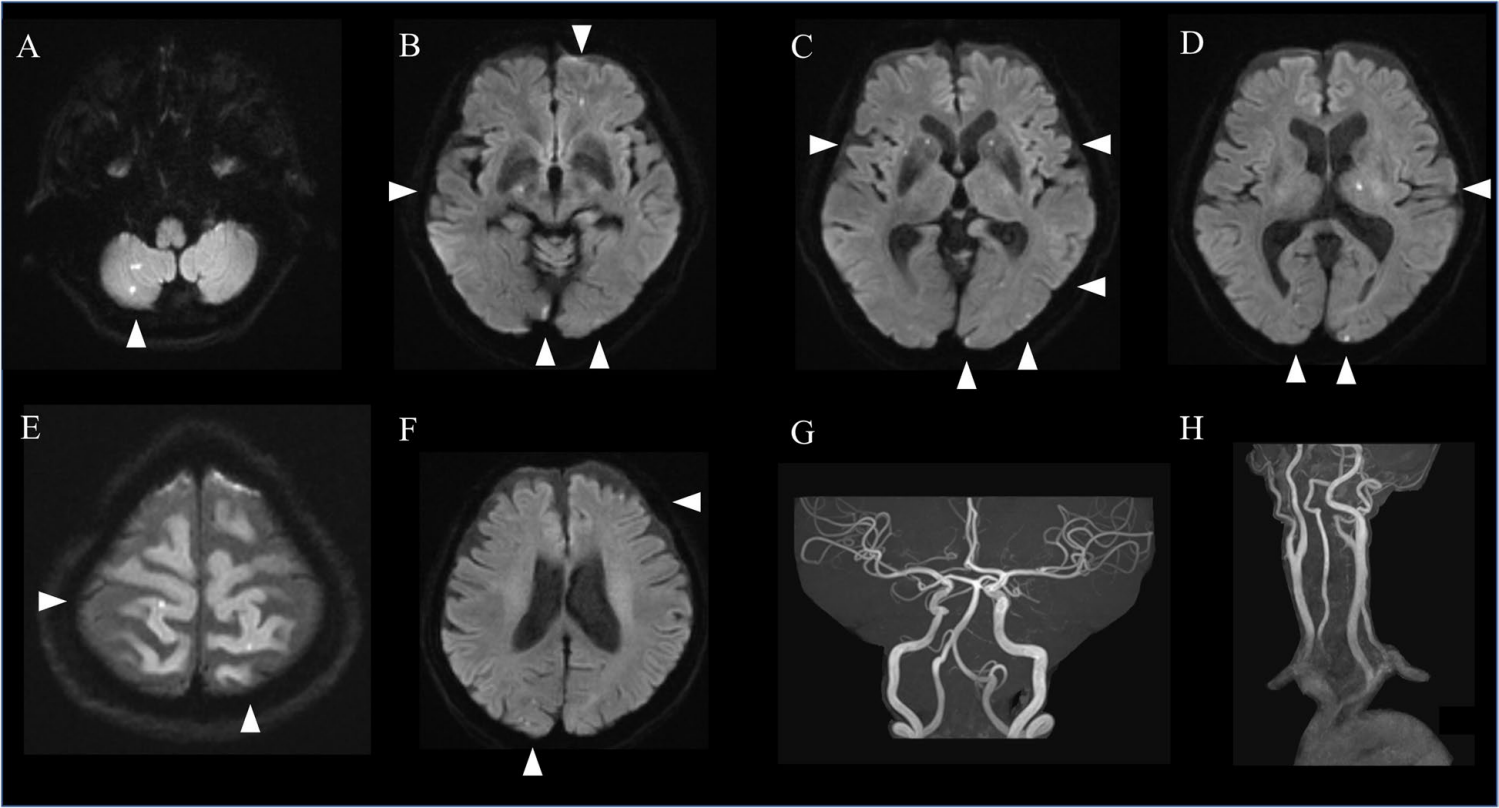
Brain magnetic resonance images on diffusion-weighted imaging on admission. Multiple small lesions, which showed high intensity on diffusion-weighted imaging, are seen on the right cerebellum, left thalamus and frontal lobe, bilateral parietal lobe, occipital lobe, and nucleus basalis (**A**-**F**). Magnetic resonance angiography was unremarkable (**G** and **H**)

The patient was then presented to our hospital for workup. Neurologic examination confirmed bilateral hearing loss, which was dominant in the left. She had no general signs and symptoms of vasculitis. Brain magnetic resonance imaging (MRI) revealed acute-stage multiple cerebral infarctions on diffusion-weighted imaging sequence (Fig. 2A-F). Magnetic resonance angiography (Fig. 2G and H) and computed tomographic angiography demonstrated regular vascular walls; they showed no luminal narrowing or obstruction of the cerebral arteries, or no other embolic lesions. A whole body computed tomography was unremarkable. Laboratory findings showed D-dimer elevated to 1.51 μg/mL and C-reactive protein to 1.39 mg/dL, whereas a white blood cell count was 8400 /μL, blood urea nitrogen 15.0 mg/dL, and serum creatinine 0.77 mg/dL all within the normal range. All the other laboratory findings, including those on calcium and phosphate metabolisms, blood coagulation system such as hypercoagulation, antibodies associated with vasculitis, and common tumor markers, were unremarkable. TTE and transesophageal echocardiography showed no vegetations or thrombi but revealed an immobile hyperechoic 14.9 × 12.1 mm mass (Fig. 1B), which was suspected as a CAT. The mass had an acoustic shadow arising from a posterior cusp of the mitral valve with MAC. Carotid artery ultrasound and Holter electrocardiography were unremarkable. Repeated blood cultures during hospitalization were negative. Mini-mental status examination score was 29/30. The past medical history, besides TIA, included Ménière's disease and hypertension; there was no history of hyperlipemia, diabetes mellitus, atrial fibrillation, or psychiatric diseases.

**Fig. 3:**
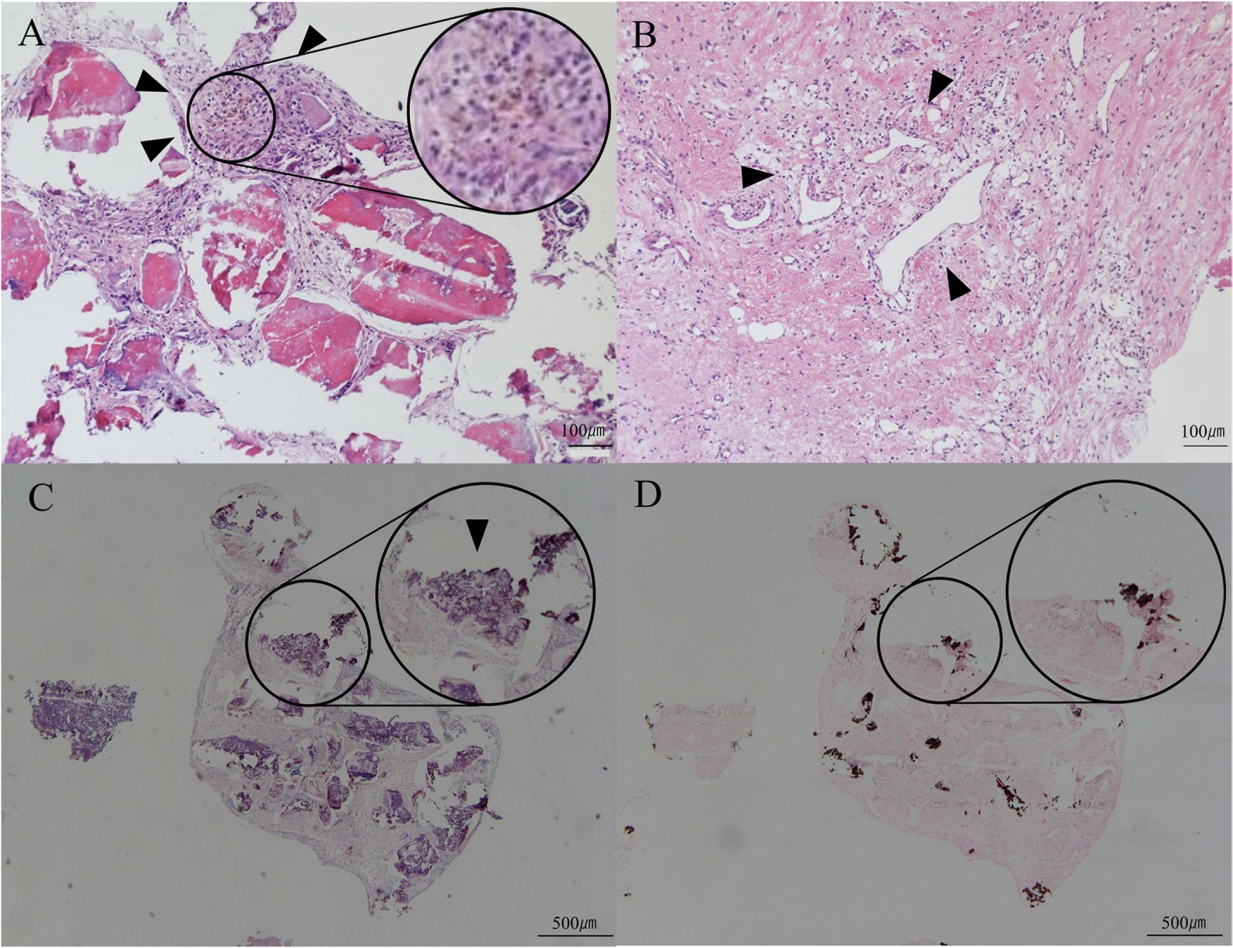
Pathological images of the resected tissue. Amorphous fibrous tissue surrounding the multiple nodular calcifications, lymphocyte infiltration and hemosiderin deposition were confirmed in the fibrous tissue (**A**, arrowheads). Neoangiogenesis occured in the mitral valve (**B**, arrowheads). CAT was stained by phosphotungstic acid-hematoxylin (PTAH) stain (**C**) and Von Kossa stain (**D**). Fibrin around CAT and calcification of CAT were shown in blue/purple by PTAH stain (**C**). Calcification of CAT was shown in black/brown by Von Kossa stain (**D**). In comparing (**C**) with (**D**), the area that was stained in (**C**) but not in (**D**) indicated fibrin attached to CAT (**C**, arrowhead)

The patient was admitted for the treatment of acute multiple cerebral infarctions, and apixaban 5 mg/day and aspirin 100 mg/day were administered. On the 9 th day of admission, follow-up MRI showed several newly-developed cerebral infarctions; at the same time, follow-up TTE showed the mass from the mitral valve diminished to 13.8 × 9.7 mm (Fig. 1C). The degree and frequency of the musical hallucination decreased and returned to those before developing the fever. 1 month after the admission, a surgical resection of the mass and replacement of mitral valve by bioprosthetic valve was performed. Intraoperatively, a calcified mass was confirmed at the posterior leaflet, along with MAC and an additional subvalvular lesion. The pathological findings showed calcifications surrounded by amorphous fibrous tissue, including fibrin; lymphocyte infiltration and hemosiderin deposition in the fibrous tissue; and neoangiogenesis in the mitral valve. They showed no evidence of valve destruction or previous infective endocarditis (Fig. 3A-D). Four months after the infarctions, follow-up brain MRI revealed no further infarctions.

## Discussion and conclusions

CAT of the heart is a rare non-neoplastic cardiac tumor [1], and sometimes causes embolism. Herein, we reported a first case of CAT that associated with an antecedent infection and caused multiple cerebral infarctions in an 82-year-old Japanese woman. The case was also first in the sense that the CAT not only grew rapidly but also diminished rapidly in a short period of time. In addition, unlike many of the CAT patients in the previous reports, the patient had normal renal function.

CAT may be associated with infection, but only 2 such cases have been reported [4, 5]. Still there has been no case reported of antecedent infection until our report. In our case, the patient had an infection from a dental treatment, and then developed multiple cerebral infarctions from CAT, indicating that infection was not a complication of CAT in our case. As a mechanism underlying infarction, CAT itself or the fibrin on the mass surface is considered to be the source of clogging [10]. Meanwhile, CAT can grow rapidly from 6 weeks to 1 year [6–9]. In our case, CAT grew in 7 months and caused multiple infarctions. Notable in our case was that we observed not only a rapid growth but also a rapid shrinkage of CAT after the infarctions. Also notable was that, while the patients were on hemodialysis in most cases of rapidly growing CAT [6, 7, 9], our patient was normal in renal function.

Reportedly, factors that may contribute to rapid growth of CAT include abnormal calcium metabolism due to renal dysfunction [6], inflammation associated with hemodialysis [7], and mobile CAT arising from MAC [8]. None of these factors were found in our case; instead lymphocyte infiltration, hemosiderin deposition, fibrin attached to the surface of CAT, and neoangiogenesis were observed, all of which suggested inflammation. We speculate that inflammation may trigger formation of CAT: inflammation from the infection caused coagulation system activation, had fibrin form around the surface of CAT, and lead to rapid growth of CAT, resulting in acute-stage cerebral infarctions in 1 month. Similar process could have happened in some cases of rapid growing CAT.

In our case, CAT was thought to be a cause of cerebral infarctions, as no other possible causes were found. The follow-up TTE showed the diminished CAT, and the brain MRI on the same day revealed newly-developed multiple cerebral infarctions, suggesting a strong association of CAT with the infarctions. Supposedly, CAT may cause infarction by CAT itself that comes off of the heart or fibrin on the mass surface that also comes off of the surface [10]. In previous case where CAT itself was thought to come off, an intravascular thrombus with calcification was typically observed [11]. Such a thrombus was not, however, found on the brain computed tomography in our case. In our case, the pathological findings showed fibrous tissue and fibrin, which could easily collaps. Taken together, fibrin on the mass, rather than CAT itself, appeared a more likely cause of the infarctions. At the same time, CAT can diminish by antithrombotic drugs. Aspirin 100 mg/day was reported to result in diminised and then disappeared CAT in 2 months [12]. In our case, too, drug administration may contribute to the shrinkage of CAT to some extent.

Most CAT with cerebral infarctions were mobile, mobile CAT from a MAC carry a high embolic risk [8]. However, CAT with MAC was immobile in our case. Considering that CAT caused multiple cerebral infarctions in our case, it would be better to perform a surgical operation against CAT even in the absence of high embolic risks.

In summary, we reported a case of multiple cerebral infarctions from CAT associated with the inflammation by the antecedent infection following dental therapy. In this case, fibrin attaching to the CAT appeared to cause the CAT to grow rapidly, leading to multiple cerebral infarctions under antithrombotic drugs. Fibrous tissue including fibrin around the CAT was thought to disappear quickly by coming off of the surface and by antithrombotic drugs, leading to the rapid decrease of CAT in size. Our case showed that CAT can grow rapidly by an antecedent infection in a patient with normal renal function and cause cerebral infarctions.

## Data Availability

All data related to this case report are documented within this manuscript.

## Abbreviations

CAT: Calcified amorphous tumor
MAC: Mitral annular calcification
TIA: Transient ischemic attack
TTE: Transthoracic echocardiography
MRI: Magnetic resonance imaging
